# Upregulation of Matrix Metalloproteinase-9 in Primary Cultured Rat Astrocytes Induced by 2-Chloroethanol Via MAPK Signal Pathways

**DOI:** 10.3389/fncel.2017.00218

**Published:** 2017-07-19

**Authors:** Tong Wang, Yingjun Liao, Qi Sun, Hongge Tang, Gaoyang Wang, Fenghong Zhao, Yaping Jin

**Affiliations:** ^1^Department of Occupational and Environmental Health, School of Public Health, China Medical University Shenyang, China; ^2^Department of Physiology, China Medical University Shenyang, China

**Keywords:** 2-chloroethanol, astrocytes, mitogen-activated protein kinase signal pathway, matrix metalloproteinases-9, 1, 2-dichloroethane poisoning

## Abstract

2-Chloroethanol (2-CE) is one of the reactive metabolites of 1,2-DCE *in vivo*, which might contribute to brain edema formation induced by 1,2-dichloroethane (1,2-DCE) poisoning. Thus, the purpose of this study was to explore the roles of mitogen-activated protein kinase (MAPK) signal pathways in upregulation of matrix metalloproteinase-9 (MMP-9) in 2-CE exposed rat astrocytes. Expression of p38 MAPK (p38), extracellular signal regulated protein kinase (ERK), c-Jun N-terminal kinase (JNK) and MMP-9 at both protein and gene levels in rat astrocytes were determined using western blot and real-time RT-PCR methods. The results showed that both protein and mRNA levels of MMP-9 in 2-CE exposed astrocytes significantly increased. Meanwhile, protein levels of phosphorylated p38 (p-p38), ERK1/2 (p-ERK1/2) and JNK1/2 (p-JNK1/2) in 2-CE exposed astrocytes also significantly increased. In addition, both protein and mRNA levels of MMP-9 significantly decreased in response to reduced protein levels of p-p38, p-ERK1/2 and p-JNK1/2 achieved by supplement with their specific inhibitors, indicating that activation of MAPK signal pathways might play an important role in upregulation of MMP-9 expression at the transcriptional level in 2-CE exposed astrocytes. Furthermore, since pretreatment of n-acetyl-l-cysteine (NAC), a powerful antioxidant amino acid, could attenuate the elevated levels of MMP-9, p-p38, p-ERK2 and p-JNK1/2 in 2-CE exposed astrocytes, activation of MAPK signal pathways in 2-CE exposed astrocytes could be mediated partially by reactive oxygen species (ROS), which was most likely generated in the metabolism of 2-CE.

## Introduction

1,2-Dichloroethane (1,2-DCE, CAS number: 107-06-2) is a synthetic organic chemical that is mainly used for production of vinyl chloride. It is normally utilized as the organic solvent, especially as the thinner of adhesives (Sun et al., 2016). Accumulated evidence has demonstrated that toxic encephalopathy caused by 1,2-DCE poisoning has become one of the serious occupational diseases in China (Liu et al., 2010; Sun et al., 2015). Brain edema is deemed to be the main pathological processes involved in the toxic encephalopathy. However, the molecular mechanisms of brain edema induced by 1,2-DCE poisoning remains unknown.

Abundant experimental studies have demonstrated that the main metabolic pathway of 1,2-DCE *in vivo* is mediated by microsomal cytochrome P450 2E1 (CYP2E1; Reitz et al., 1982; Sweeney et al., 2008). The metabolic intermediates produced in CYP2E1 mediated 1,2-DCE metabolism are 2-chloroethanol (2-CE) and chloroacetaldehyde, which are proposed to be involved in the toxic effects induced by 1,2-DCE poisoning, because they are more reactive than 1,2-DCE itself (Guengerich et al., 1980; Igwe et al., 1986; Raucy et al., 1993). Therefore, it is indispensable for exploring the neurotoxicity directly caused by 2-CE. On the other hand, since CYP2E1 has an intense NADPH oxidase activity compared to several other CYP450 isoforms, more reactive oxygen species (ROS) can be generated in the metabolic course (Liu et al., 2005).

Brain edema is a life threatening disease, which may lead to intracranial hypertension due to either extracellular or intracellular accumulation of abnormal fluid. The former is called as vasogenic brain edema and due to breakdown of blood-brain barrier (BBB), which allows passage of plasma proteins and water into the extracellular compartment, while the latter called as cytotoxic brain edema is due to excessive intake of water by injured brain cells (Simard and Rivest, 2007). Recently, the roles of matrix metalloproteinases (MMPs) in the pathogenesis of vasogenic edema have attracted great attention.

MMPs are the family of zinc-dependent endopeptidases consisting of more than 20 members. They are known to be related to degradation of component in extracellular matrix (ECM) and vascular basement membrane (Sternlicht and Werb, 2001). As the main gelatinases, MMP-2 and MMP-9 can degrade the collagen IV and fibronectin in the basal lamina around capillaries, therefore, overexpression of MMP-2 and MMP-9 will lead to development of vasogenic brain edema (Asahi et al., 2001a,b; Ramos-Fernandez et al., 2011). A variety of studies have demonstrated that MMP-9 expression is enhanced in animal models of cerebral ischemia, hemorrhage, and trauma (Planas et al., 2002; Sumii and Lo, 2002; Wang et al., 2002), which are attributed to breakdown of BBB (Planas et al., 2001; Ramos-Fernandez et al., 2011). Our recent studies also demonstrated that mRNA levels of MMP-9 in the cerebral tissues of 1,2-DCE poisoned mice significantly increased at the early phase of brain edema formation (Wang G. et al., 2014). Accordingly, we hypothesized that upregulation of MMP-9 expression may play the key roles in the pathological processes of brain edema formation induced by 1,2-DCE poisoning. However, the regulatory mechanisms for MMP-9 upregulation during 1,2-DCE induced brain edema formation are unclear.

The mitogen-activated protein kinase (MAPK) signal pathways comprise three main groups of kinase, which are extracellular signal related kinases (ERKs), p38 MAPKs (p38) and c-Jun amino terminal kinases (JNKs; Tejima et al., 2007; Hsieh et al., 2010; Ralay Ranaivo et al., 2011). These intracellular signal pathways transport extracellular signals into the nucleus in response to multi-stimuli. Therefore, they play the pivotal roles in many essential cellular processes, and are considered as the important regulators in pathogenesis of diseases. Activation of these signal pathways is through phosphorylation by upstream kinases. After activation of these signal pathways, a variety of MAPK-regulated transcription factors are activated, which may lead to expression of a series of target genes and result in the corresponding biological responses, including upregulation of inflammatory proteins, such as MMP-9, tumor necrosis factor-α (TNF-α) and interleukin-1β (IL-1β; Hsieh and Papaconstantinou, 2004; Wu et al., 2004). It has been reported that in response to suppressed MMP-9 expression in the brain achieved by the specific MAPK inhibitor, the concomitant edema and cortical injury in mice after brain trauma were ameliorated (Mori et al., 2002), suggested that MAPK signal pathways can play the key roles in modulation of MMP-9 expression.

As the most numerous cell type in the brain, astrocytes perform many functions, such as formation of the BBB, provision of nutrients to the nervous tissue and maintenance of homeostatic balance (Janzer and Raff, 1987; Liberto et al., 2004; del Zoppo and Milner, 2006). It is known that astrocytes can be activated in response to different external stimuli, and then secrete diverse proinflammatory cytokines, such as MMP-9, TNF-α and IL-1β. Recent studies have disclosed that activation of ERK1/2 and p38 can contribute to lipopolysaccharide (LPS) induced upregulation of MMP-9 expression in the astrocytes (Hsieh and Papaconstantinou, 2004; Wu et al., 2004). Taken together, the purpose of present study was to explore the regulatory mechanisms of MMP-9 expression through MAPK signal pathways in 2-CE exposed rat astrocytes. Since overexpression of MMP-9 can result in brain damage, studying the regulatory pathways of MMP-9 expression is necessary for identifying new therapeutic targets in the treatment of 1,2-DCE induced brain edema. As far as we know, the literature on this aspect has not been reported.

## Materials and Methods

### Animal Care and Use Statement

All animal studies were provided by the Scientific Research Committee of China Medical University and was conducted in accordance with Chinese National Guidelines for the Protection of Laboratory Animal in animal experiments.

### Reagents

2-CE with purity more than 99.0% was obtained from the Sinopharm Chemical Reagent Co., Ltd, China. Dulbecco’s modified Eagle’s medium (DMEM), trypsin, penicillin and streptomycin were the products of Invitrogen (USA). Heat inactivated fetal bovine serum (FBS) was obtained from Biological Industries (Israel). The enhanced chemiluminescence (ECL) plus kit and bicinchoninic acid (BCA) protein assay kit were the products of Pierce (Thermo Fisher Scientific, USA). RIPA Lysis Buffer was purchased from Beyotime Biotechnology (China). SB202190, U0126, and SP600125 were purchased from Selleck (USA). Polyclonal antibodies against MMP-9 (^#^AB19016) and GFAP (^#^MAB360) were obtained from Millipore (USA). Antibodies of phosphorylated p38 (p-p38, ^#^9211), ERK1/2 (p-ERK1/2, ^#^4370), and JNK1/2 (p-JNK1/2, ^#^9251), as well as p38 (^#^9212), ERK1/2 (^#^4695), JNK1/2 (^#^9258) and β-actin (^#^4970) were the products of Cell Signaling Technology (USA). Secondary antibody conjugated fluorescein isothiocyanate (FITC) and tetraethyl rhodamine isothiocyanate (TRITC) were obtained from the Beyotime Biotechnology (China). All reagents used in present study were of the analytical grade and provided by the local chemical suppliers.

Reagents listed above were prepared as stock solutions with sterile water, and then diluted to the target concentration before use. Water used in present study was doubly distilled.

### Primary Culture for Astrocyte

Primary culture for astrocyte was prepared as the procedure described previously (Sun et al., 2017). Briefly, the cerebral cortices of both sexes from postnatal day 1–3 Wistar rats were cautiously separated, and then rinsed three times by ice-cold Hanks balanced salt solution (HBSS) without Ca^2+^ and Mg^2+^. They were chopped into pieces less than 1 mm^3^, and then digested with 0.125% (w/v) trypsin solution at 37°C for 20 min. The cell suspension was filtered through a 200-mesh stainless steel screen. After centrifugation, the dissociated cells were resuspended in DMEM containing 10% FBS and 1% penicillin-streptomycin. The cells were plated in the culture dishes pre-coated with poly-L-lysine (BD Biosciences, USA) at a density of 1 × 10^6^/cm^2^, and then incubated at 37°C, 5% CO_2_ and 100% humidified atmosphere. During incubation, the medium was changed every 3 days. When a confluent layer was formed, the culture dishes were placed on a horizontal shaker, and shaken at 250 rpm for 15 h to remove oligodendrocyte precursors and microglia. After shaking, the culture medium was replaced immediately, and a nearly pure astrocyte was remained in the culture dishes. The target cells were grown for 24 h, and then resuspended by trypsin and reseeded at a density of 1 × 10^5^/cm^2^.

### Treatments

The stock solution of 1 M (mol/L) 2-CE was prepared with redistilled water, and then diluted to the final concentrations by DMEM containing 5% FBS before use. The experiment in this study was divided into three parts. In the first part, when a confluent layer was formed, astrocytes were exposed to 0, 7.5, 15 and 30 mM of 2-CE for 24 h in DMEM with 5% FBS. After exposure, the cells were collected for analysis of MMP-9 expression at both protein and gene levels. In the second part, the cells were divided into seven groups to investigate the roles of MAPK signal pathways in upregulation of MMP-9 expression. They were control groups (including blank control, solvent control and inhibitor control), exposure group, and intervention groups (treated with low, middle and high dose of inhibitor), respectively. Cells in the solvent control were exposed to dimethyl sulfoxide (DMSO), which was less than 0.1% in the medium. Cells in the inhibitor control were exposed to 30 μM of SB202190, 10 μM of U0126 or 10 μM of SP600125. The inhibitors were dissolved with DMSO, and then added into the medium (Wu et al., 2004; Sun et al., 2017). Cells in the exposure group were exposed to 30 mM of 2-CE for 24 h. Cells in the intervention groups were pretreated with 1, 10 and 30 μM of SB202190 for inhibition of p38 (Manthey et al., 1998), 0.1, 1 and 10 μM of U0126 for inhibition of ERK1/2 (Smutny et al., 2014), or 0.1, 1 and 10 μM of SP600125 for inhibition of JNK1/2 (Bennett et al., 2001). One hour later, the cells were exposed to 30 mM of 2-CE for 24 h. At the end of exposure, the cells were collected for detecting expression of MMP-9, p38, ERK1/2 and JNK1/2, as well as phosphorylated p38 (p-p38), ERK1/2 (p-ERK1/2) and JNK1/2 (p-JNK1/2) at both protein and gene levels. In the third part, to explore the effects of n-acetyl-l-cysteine (NAC), the scavenger of peroxide, on generation of ROS and activation of MAPK signal pathways induced by 2-CE, the cells were divided into five groups, which were control group, exposure group and intervention groups (treated with low, middle and high dose of NAC), respectively. Cells in the intervention groups were pretreated with 5, 50 and 500 μM of NAC in the medium. One hour later, the cells were exposed to 30 mM of 2-CE for 24 h. At the end of exposure, the cells were collected for analysis of intracellular ROS levels and protein levels of MMP-9, p-p38, p-ERK1/2 and p-JNK1/2.

### Analysis

#### Immunofluorescence Staining

Astrocytes were washed three times with PBS, fixed with 4% paraformaldehyde, and then permeabilized in 0.2% Triton X-100. The nonspecific binding of antiserum was blocked with 10% normal goat serum at room temperature. They were then incubated with primary antibodies against MMP-9 (1:100, rabbit anti-rat) and GFAP (1:200, mouse anti-rat) at 4°C overnight. Labeled cells were visualized by secondary antibodies conjugated FITC (goat anti-rabbit) and TRITC (goat anti-mouse), and observed with the fluorescence microscope (Olympus IX70). Digital images were obtained by a digital camera system (Olympus SC35). For negative controls, the primary antibodies were omitted.

#### Intracellular ROS Assay

The dichlorofluorescein diacetate (DCF-DA) obtained from Beyotime Biotechnology (China), was used to assess the levels of intracellular ROS. Briefly, after exposure to 2-CE, the cells were incubated with serum-free medium supplemented with 5 μM DCF-DA at 37°C for 30 min, and then washed with PBS twice. The relative fluorescence intensity was recorded by a fluorescent microplate reader (BioTek Instruments, Inc., Winooski, VT, USA) at an excitation wavelength of 485 nm and emission wavelength of 535 nm. Results were reported with respect to unexposed cells.

#### Western Blot Analysis

After exposure, the cells were rinsed with cold PBS, detached by scraping, and then harvested by centrifugation for 5 min at 1500× *g*. Cell pellets were suspended and lysed by RIPA buffer. Lysate was centrifuged at 4°C, 12,000× *g* for 10 min. Protein concentrations were determined by the BCA protein assay kit. The samples of total protein (40 μg per lane) were loaded to 10% SDS-PAGE, and then resolved by electrophoresis. Blots were transferred onto polyvinylidene difluoride (PVDF) membranes (Millipore, USA), and then probed with the rabbit anti-rat primary antibodies against MMP-9, p-p38 (Thr^180^/Tyr^182^, 1:500), p38 (1:1000), p-ERK1/2 (Thr^202^/Tyr^204^, 1:500), ERK1/2 (1:1000), p-JNK1/2 (Thr^183^/Tyr^185^, 1:500), JNK1/2 (1:1000) and β-actin (1:2000) at 4°C overnight. Bands of target protein were detected by the secondary antibodies (1:5000), and visualized by the ECL plus kit. The integrated intensity of each band was semi-quantitatively assessed by densitometry using the image analyzing software (Gel-Pro analyzer v4.0). It was normalized to β-actin (served as the internal standard) from the same blot.

#### Quantitative Real-Time RT-PCR

Total RNA was extracted from astrocytes with Trizol Reagent, and the first strand of cDNA was generated by RT-PCR using the PrimeScript RT reagent kit (Takara, Japan). Subsequently, the cDNA was served as templates for real-time PCR amplification using the SYBR Premix Ex Taq II (Takara, Japan) and ABI 7500 Real-Time PCR System (Applied Biosystems, USA). To amplify fragments of MMP-9, p38, ERK1, ERK2, JNK1, JNK2 and GAPDH (as the house-keeping gene), the following primer pairs listed in Table 1, were used. Amplification was conducted for 40 cycles of 5 s at 95°C and 34 s at 60°C. Results were analyzed using the comparative Ct method. RNA abundance were expressed as 2^−ΔΔCt^ for the target gene normalized against the GAPDH, and presented as fold change vs. contralateral control samples (Yu et al., 2015).

**Table 1:** Oligonucleotide sequences used for real-time RT-PCR.

Gene	Primer (5′-3′)		Product (bp)
MMP-9	Sense	*ATCCGCAGTCCAAGAAGATT*	251
	Antisense	*GCCAGAGAACTCGTTATCCA*	
p38	Sense	*CCGAGCGATACCAGAACCT*	208
	Antisense	*AACACATCCAACAGACCAATCA*	
ERK1	Sense	*TCATAGGCATCCGAGACATCC*	237
	Antisense	*CGCAGGTGGTGTTGATAAGC*	
ERK2	Sense	*ACCGTGACCTCAAGCCTTC*	237
	Antisense	*GCCTGTTGGATAGCATCTCTG*	
JNK1	Sense	*TGGAGGAGCGAACTAAGAATGG*	103
	Antisense	*TTGACAGACGGCGAAGAGAC*	
JNK2	Sense	*ACACCATCCGCAGAGTTCAT*	222
	Antisense	*CAAGGCTTCGTCCACAGAGA*	
GAPDH	Sense	*GCAAGAGAGAGGCCCTCAG*	74
	Antisense	*TGTGAGGGAGATGCTCAGTG*	

#### Statistical Analysis

Data were expressed as mean ± standard deviation (SD), and analyzed using the SPSS for Windows, version 20.0 (SPSS Inc., Buffalo, NY, USA). Significant differences among group means were evaluated by analysis of variance test (one-way ANOVA). *Post hoc* tests were analyzed by Student-Newman-Keuls test (SNK). The statistical significance was defined as *p* < 0.05.

## Results

### Expression of MMP-9 in Rat Astrocytes Affected by Exposure to 2-CE

**Figure 1:**
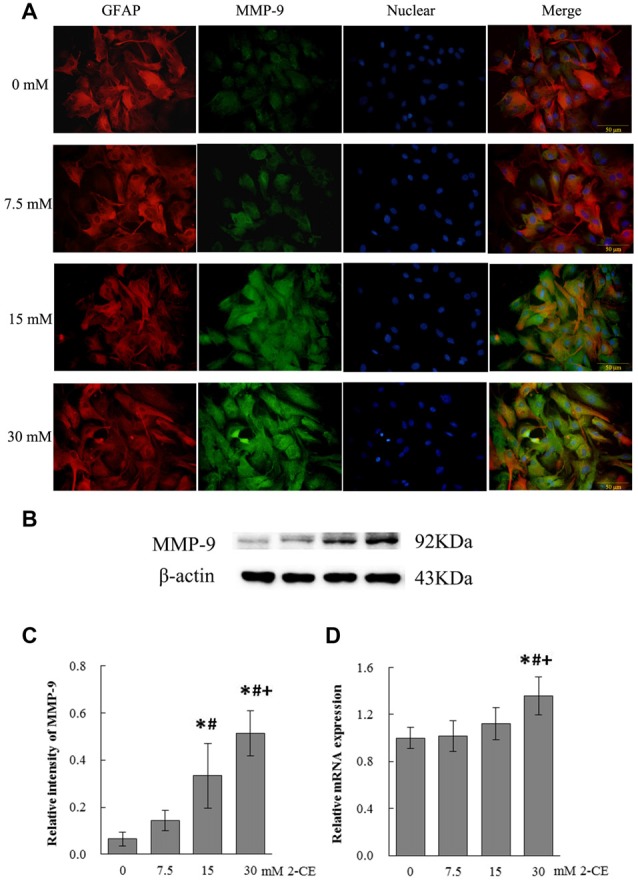
Changes of matrix metalloproteinase-9 (MMP-9) protein and mRNA levels in primary cultured astrocytes exposed to 2-chloroethanol. Notes: 2-CE: 2-chloroethanol. **(A)** Immunofluorescence staining for MMP-9 and GFAP (400×). Astrocytes grown on the 6-well plate were stained with antibody of MMP-9 conjugated FITC and GFAP conjugated TRITC. **(B)** Western blot analysis. Images were the representative results of four separate experiments for each group. **(C)** Densitometric analysis of western blots for MMP-9. The relative intensity in arbitrary units compared to β-actin. **(D)** Quantitation of MMP-9 mRNA by real-time RT-PCR. The gene expression was normalized to GAPDH and presented as fold change vs. the control. Data were expressed as mean ± standard deviation (SD) of four independent experiments performed on four batches of primary cultured astrocytes for each level of 2-CE, and analyzed by One-way ANOVA. Significant difference was defined as *p* < 0.05, and ^*^, vs. control; ^#^, vs. 7.5 mM dose; ^+^, vs. 15 mM dose.

Representative micrographs in Figure 1A showed immunoreactivity to MMP-9 in the cells captured at the end of exposure. Fluorescence intensity in 15 mM and 30 mM of 2-CE exposure groups increased apparently as compared to the control group. Photos shown in Figure 1B revealed the typical western blots for MMP-9 in the cells from different groups. Graph shown in Figure 1C disclosed quantitative analysis of western blots. Consistent with the changes of immunoreactivity, protein levels of MMP-9 in 15 mM and 30 mM of 2-CE exposure groups increased significantly as compared to the control and 7.5 mM of 2-CE exposure group. Moreover, the protein level in 30 mM of 2-CE exposure group also increased significantly compared to 15 mM of 2-CE exposure group. Graph shown in Figure 1D disclosed mRNA levels of MMP-9 affected by 2-CE exposure, and indicated those in 30 mM of 2-CE exposure group increased significantly compared to the other groups. Accordingly, exposure to 30 mM of 2-CE could confirmatively upregulate MMP-9 expression at both protein and gene levels in rat astrocytes, therefore this dosage of 2-CE was used in the next experiments.

### Roles of p38 in Upregulation of MMP-9 Expression in 2-CE Exposed Rat Astrocytes

**Figure 2:**
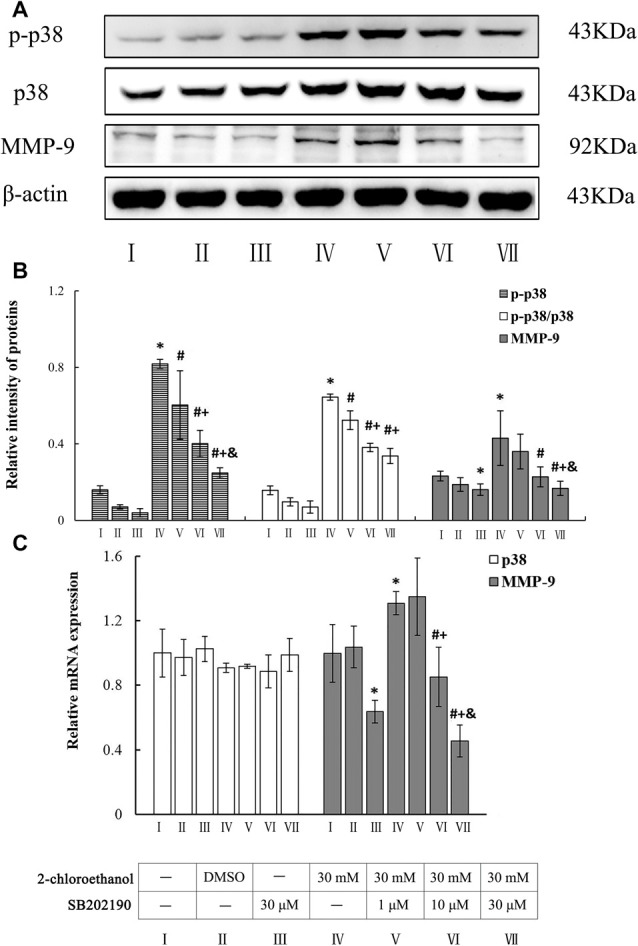
Involvement of p38 mitogen-activated protein kinase (MAPK) in MMP-9 induction in primary cultured astrocytes exposed to 2-chloroethanol. Notes: SB, SB202190; 2-CE, 2-chloroethanol. Cells in the exposure group and intervention groups were exposed to 30 mM 2-CE for 24 h. Cells in the intervention groups were pre-exposed with SB 1 h before 2-CE exposure. **(A)** Western blot analysis. Images were the representative results of four separate experiments for each group. **(B)** Densitometric analysis of western blots. The relative intensity of MMP-9 and p-p38 in arbitrary units was compared to β-actin. The ratios of phosphorylated p38 to native p38 was expressed as the p-p38/p38. **(C)** Quantitation of mRNA by real-time RT-PCR. The gene expression was normalized to GAPDH and presented as fold change vs. the control. Data were expressed as mean ± SD of four independent experiments performed on four batches of primary cultured astrocytes, and analyzed by One-way ANOVA. Significant difference was defined as *p* < 0.05, and ^*^, vs. blank control; ^#^, vs. 30 mM 2-CE exposure group; ^+^, vs. 1 μM SB inhibition group; ^&^, vs. 10 μM SB inhibition group.

Photos shown in Figure 2A demonstrated the typical western blots for p-p38, p38 and MMP-9 in the cells from different groups. Graphs shown in Figures 2B,C revealed the comparison of protein and mRNA levels among groups. As described above, both protein and mRNA levels of MMP-9 in exposure group increased apparently compared to the control group. Moreover, protein levels of p-p38 and ratios of phosphorylated p38 to native p38 (p-p38/p38) in exposure group also increased significantly compared to blank control. On the other hand, protein levels of p-p38 and p-p38/p38 in all intervention groups decreased significantly as compared to exposure group. Furthermore, compared to exposure group, both protein and mRNA levels of MMP-9 in middle and high intervention groups also decreased significantly and dose dependently in response to the changes of p-p38 protein expression. However, mRNA levels of p38 failed to show any significance among different groups.

### Roles of ERK in Upregulation of MMP-9 Expression in 2-CE Exposed Rat Astrocytes

**Figure 3:**
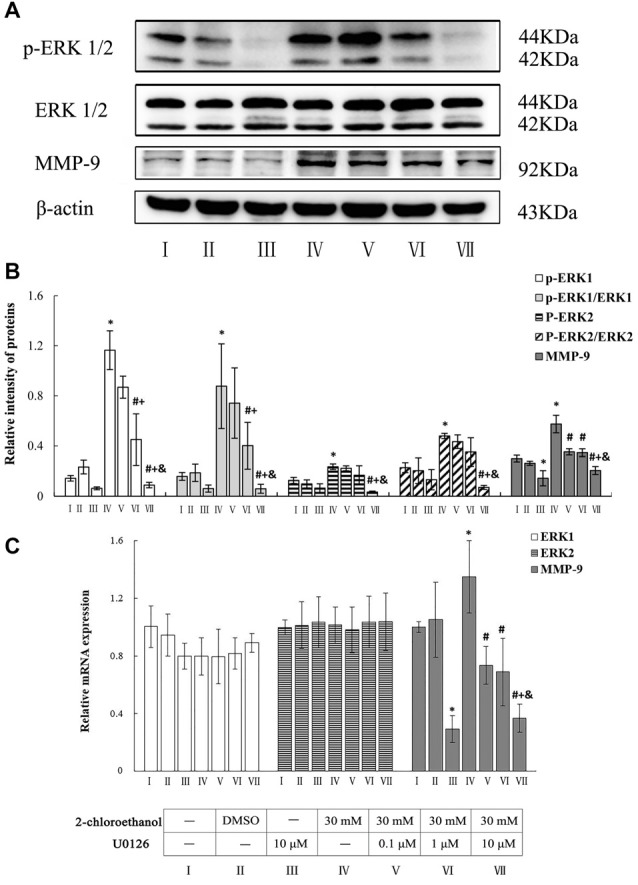
Involvement of ERK1/2 in MMP-9 induction in primary cultured astrocytes exposed to 2-chloroethanol. Notes: U0, U0126; 2-CE, 2-chloroethanol. Cells in the exposure group and intervention groups were exposed to 30 mM 2-CE for 24 h. Cells in the intervention groups were pre-exposed with U0 1 h before 2-CE exposure. **(A)** Western blot analysis. Images were the representative results of four separate experiments for each group. **(B)** Densitometric analysis of western blots. The relative intensity of MMP-9 and p-ERK1/2 in arbitrary units was compared to β-actin. The ratios of phosphorylated ERK1/2 to native ERK1/2 was expressed as the p-ERK1/ERK1 and p-ERK2/ERK2. **(C)** Quantitation of mRNA by real-time RT-PCR. The gene expression was normalized to GAPDH and presented as fold change vs. the control. Data were expressed as mean ± SD of four independent experiments performed on four batches of primary cultured astrocytes, and analyzed by One-way ANOVA. Significant difference was defined as *p* < 0.05, and ^*^, vs. blank control; ^#^, vs. 30 mM 2-CE exposure group; ^+^, vs. 0.1 μM U0 inhibition group; ^&^, vs. 1 μM U0 inhibition group.

Photos shown in Figure 3A revealed the typical western blots for p-ERK1/2, ERK1/2 and MMP-9 in the cells from different groups. Graphs shown in Figures 3B,C disclosed the comparison of both protein and mRNA levels among groups. Compared to control group, both protein and mRNA levels of MMP-9 in exposure group increased significantly. Moreover, protein levels of p-ERK1/2, ratios of phosphorylated ERK1 to native ERK1 (p-ERK1/ERK1) and ratios of phosphorylated ERK2 to native ERK2 (p-ERK2/ERK2) in exposure group also increased significantly. On the other hand, compared to exposure group, protein levels of p-ERK1 and p-ERK1/ERK1 in middle and high intervention groups decreased significantly and dose dependently, but protein levels of p-ERK2 and p-ERK2/ERK2 only in high intervention groups decreased significantly. Furthermore, in response to changes of p-ERK1/2 protein expression, both protein and mRNA levels of MMP-9 in all intervention groups decreased significantly as compared to exposure group. However, mRNA levels of ERK1 and ERK2 failed to show any significance among different groups.

### Roles of JNK in Upregulation of MMP-9 Expression in 2-CE Exposed Rat Astrocytes

**Figure 4:**
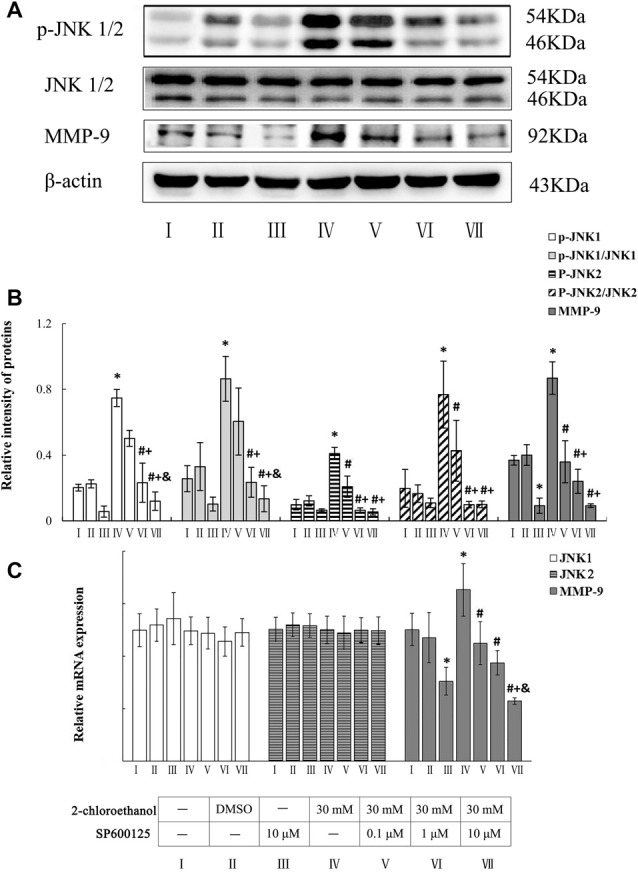
Involvement of JNK1/2 in MMP-9 induction in primary cultured astrocytes exposed to 2-chloroethanol. Notes: SP, SP600125; 2-CE, 2-chloroethanol. Cells in the exposure group and intervention groups were exposed to 30 mM 2-CE for 24 h. Cells in the intervention groups were pre-exposed with SP 1 h before 2-CE exposure. **(A)** Western blot analysis. Images were the representative results of four separate experiments for each group. **(B)** Densitometric analysis of western blots. The relative intensity of MMP-9 and p-JNK1/2 in arbitrary units was compared to β-actin. The ratios of phosphorylated JNK1/2 to native JNK1/2 was expressed as the p-JNK1/JNK1 and p-JNK2/JNK2. **(C)** Quantitation of mRNA by real-time RT-PCR. The gene expression was normalized to GAPDH and presented as fold change vs. the control. Data were expressed as mean ± SD of four independent experiments performed on four batches of primary cultured astrocytes, and analyzed by One-way ANOVA. Significant difference was defined as *p* < 0.05, and ^*^, vs. blank control; ^#^, vs. 30 mM 2-CE exposure group; ^+^, vs. 0.1 μM SP inhibition group; ^&^, vs. 1 μM SP inhibition group.

Photos shown in Figure 4A demonstrated the typical western blots for p-JNK1/2, JNK1/2 and MMP-9 in the cells from different groups. Graphs shown in Figures 4B,C disclosed the comparison of both protein and mRNA levels among different groups. Compared to control group, both protein and mRNA levels of MMP-9 in exposure group increased significantly. Likewise, protein levels of p-JNK1/2, ratios of phosphorylated JNK1 to native JNK1 (p-JNK1/JNK1) and ratios of phosphorylated JNK2 to native JNK2 (p-JNK2/JNK2) in exposure group also increased significantly. On the other hand, compared to exposure group, protein levels of p-JNK1 and p-JNK1/JNK1 in middle and high intervention groups decreased significantly and dose dependently, in addition, protein levels of p-JNK2 and p-JNK2/JNK2 in all intervention groups decreased significantly. Furthermore, compared to exposure group, both protein and mRNA levels of MMP-9 in all intervention groups decreased significantly in response to the changes of p-JNK1/2. But, mRNA levels of JNK1 and JNK2 failed to show any significance among different groups.

### Roles of ROS in Activation of MAPK Signal Pathways in 2-CE Exposed Rat Astrocytes

**Figure 5:**
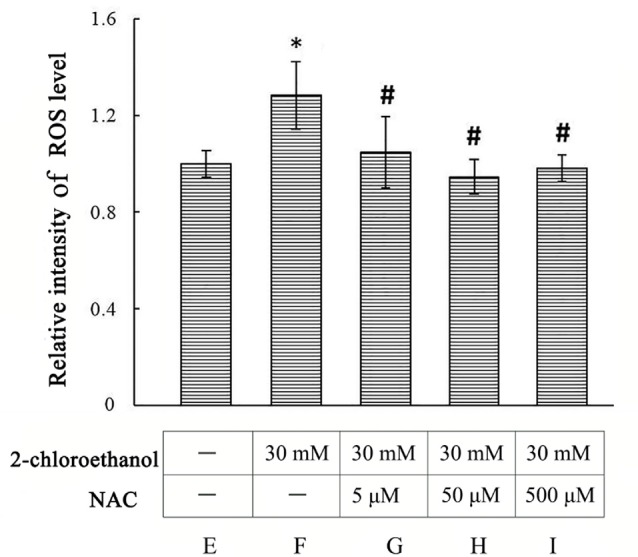
Comparison of intracellular reactive oxygen species (ROS) levels in primary cultured astrocytes among different groups Notes: 2-CE, 2-chloroethanol. Cells in the exposure group and n-acetyl-l-cysteine (NAC) intervention groups were exposed to 30 mM 2-CE. Cells in the intervention groups were pre-exposed with NAC 1 h before 2-CE exposure. Data were expressed as mean ± SD of four independent experiments performed on four batches of primary cultured astrocytes, and analyzed by One-way ANOVA. Significant difference was defined as *p* < 0.05, and ^*^, vs. blank control; ^#^, vs. 30 mM 2-CE exposure group.

**Figure 6:**
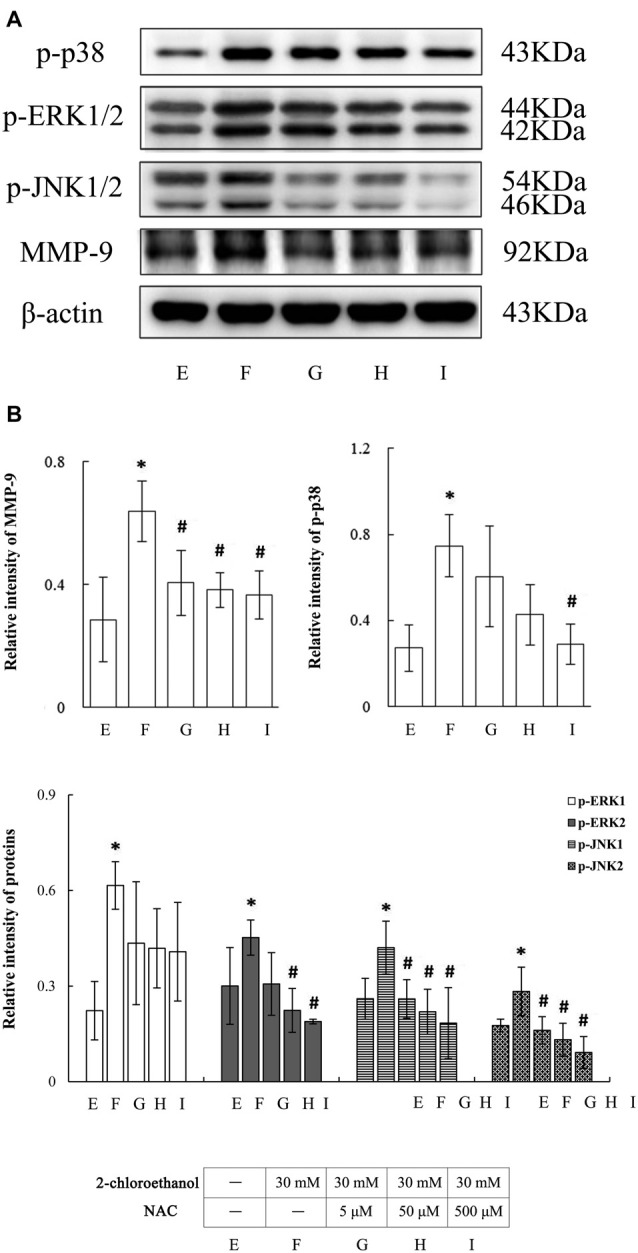
Involvement of ROS in activation of MAPK signal pathways in primary cultured astrocytes exposed to 2-chloroethanol. Notes: 2-CE, 2-chloroethanol. Cells in the exposure group and NAC intervention groups were exposed to 30 mM 2-CE. Cells in the intervention groups were pre-exposed with NAC 1 h before 2-CE exposure. **(A)** Western blot analysis. Images were the representative results of four separate experiments for each group. **(B)** Densitometric analysis of western blots. The relative intensity in arbitrary units was compared to β-actin. Bands of p-ERK1/2 or p-JNK1/2 were calculated separately. Data were expressed as mean ± SD of four independent experiments performed on four batches of primary cultured astrocytes, and analyzed by One-way ANOVA. Significant difference was defined as *p* < 0.05, and ^*^, vs. blank control; ^#^, vs. 30 mM 2-CE exposure group.

As shown in Figure 5, levels of intracellular ROS in exposure group increased significantly as compared to control group. In contrast, those in NAC intervention groups decreased significantly compared to exposure group. But, they did not differ significantly among NAC intervention groups. Photos shown in Figure 6A revealed the typical western blots for MMP-9, p-p38, p-ERK1/2 and p-JNK1/2 in the cells from different groups. Graph shown in Figure 6B disclosed quantitative analysis of western blots. Consistent with the results described above, protein levels of p-p38, p-ERK1/2 and p-JNK1/2 in exposure group increased significantly. In contrast, compared to exposure group, protein levels of p-p38 in high intervention group and p-ERK2 in both middle and high intervention groups decreased significantly. In addition, protein levels of p-JNK1/2 in the intervention groups also decreased significantly as compared to exposure group; however, they did not differ significantly among intervention groups. Furthermore, compared to exposure group, protein levels of MMP-9 in NAC intervention groups also significantly decreased in response to changes of MAPK signal pathway. But, they did not differ significantly among intervention groups.

## Discussion

To evaluate the cytotoxicity induced by 2-CE, cell viability and morphological observation of primary cultured rat astrocytes were examined at the end of exposure to 7.5, 15, 30, 60 and 120 mM of 2-CE for 24 h in our previous work. Due to increased number of detached cells, the cellular density dropped dramatically in 30, 60 and 120 mM of 2-CE exposure groups. Moreover, compared to control group, cell viability reduced significantly in 15, 30, 60 and 120 mM of 2-CE exposed groups. Based on these data, exposure of astrocyte to 7.5 mM of 2-CE did not result in any impairment on cell viability. Therefore, exposure of astrocyte to 7.5, 15 and 30 mM of 2-CE were used in this study (Sun et al., 2016).

Findings from this study suggested foremost that exposure to 2-CE, the main reactive metabolite of 1,2-DCE *in vivo*, could upregulate MMP-9 expression at transcriptional level in astrocytes. Similarly, our previous studies demonstrated that mRNA levels of MMP-9 increased markedly in the brain at the early stage of brain edema induced by 1,2-DCE in mice. These results suggested that upregulated MMP-9 expression in the brain might be resulted partially from direct effects of 2-CE in mice. Many reports have shown that excessive expression of MMP-9 could lead to brain inflammation and breakdown of BBB in the traumatic, hemorrhagic and ischemic brain injury. Thus, the generation of 2-CE *in vivo* may lead to the formation of vasogenic brain edema in mice poisoned by 1,2-DCE. Second, results of present study clearly demonstrated that exposure to 2-CE could promote protein phosphorylation of p38, ERK1/2 and JNK1/2. We also further investigated the association between upregulation of MMP-9 expression and MAPK signal pathways activation in astrocytes exposed to 2-CE by the addition of specific inhibitors. Both protein and mRNA levels of MMP-9 decreased markedly as a result of the suppressed protein levels of p-p38, p-ERK1/2 and p-JNK1/2 in 2-CE exposed astrocytes, indicating that activation of MAPK signal pathways might play important roles in upregulation the expression of MMP-9 in 2-CE exposed astrocytes. It has been well established that p38 plays the central roles in inflammation, and is strongly activated by various stress conditions and inflammatory cytokines (Borders et al., 2008; Kant et al., 2011; Yang et al., 2014). The p-p38 proteins can translocate from the cytosol into the nucleus or can transfer to the other parts of the cell and then activate the downstream kinases or several transcription factors. JNK has been shown to play the crucial roles in controlling cell proliferation and apoptosis. It is strongly activated in response to a variety of stress signals (Bas et al., 2015). The activation of JNK pathway can also result in gene transcription. ERKs in general, are pivotal regulators of proliferation and differentiation. They can be activated by many factors, and then translocate to the nucleus, where they activate various transcription factors, and ultimately leading to changes in gene expression (Fujimoto et al., 2007; Wu et al., 2010; Gladbach et al., 2014). Because the three kinds of MAPK signal pathways possess many similarities, they may undergo cross-talk at several levels. In this study, inhibition of p-p38, p-ERK1/2 and p-JNK1/2 all markedly attenuated MMP-9 expression in 2-CE exposed astrocytes, suggesting that all three kinds of MAPK signal pathways were activated, and collectively involved in modulation of MMP-9 expression in 2-CE exposed astrocytes. Until now, there is a large body of evidence to suggest that MMP-9 expression is regulated by p38, ERKs and JNKs in different cell types (Lee et al., 2014; Kim et al., 2015; Lian et al., 2015). However, as far as we know, this is the first time to discuss the molecular mechanisms of MMP-9 expression regulation in astrocytes exposed to 2-CE.

Another major finding from this study was that exposure to 2-CE could increase ROS production in rat astrocytes, and pretreatment with NAC, a scavenger of ROS, could reduce intracellular levels of ROS significantly. In response to suppressed intracellular levels of ROS, the protein levels of p-p38, p-ERK2 and p-JNK1/2 decreased markedly, suggesting that activation of MAPK signal pathways might partially mediated by increased ROS in 2-CE exposed rat astrocytes. Our previous results demonstrated that 1,2-DCE could enhance protein levels and enzymatic activity of CYP2E1, and cause oxidative damage in the liver of mice, serving as an important mechanism underlying 1,2-DCE induced toxic effects (Sun et al., 2015). Although CYP2E1 is most abundant in the liver, it also presents in many extrahepatic tissues (Sun et al., 2016). Many reports have shown that CYP2E1 is constitutively expressed in both glial cells and neurons of rat brain, and there is a correlation between expression of CYP2E1 and toxic effects of ethanol within specific brain regions (Kapoor et al., 2006). These results suggested that presence of CYP2E1 in the brain might be of physiological and toxicological importance. Recent studies have shown that ROS played the key role in upregulation of MMPs via p38 and JNK signal pathways (Ralay Ranaivo et al., 2012; Su et al., 2013; Hsieh et al., 2014). In addition, pretreatment with NAC or diphenyleneiodonium chloride (DPI), the ROS scavenger or generation inhibitor could markedly reduce the levels of MMPs and expression of MAPKs in astrocytes (Ralay Ranaivo et al., 2012; Wang J. et al., 2014). Thus, findings from this study strongly agreed with those previously reported results in this respect.

In conclusion, our findings indicated that MAPK signal pathways might be activated and involved in upregulation of MMP-9 expression at the transcriptional level in 2-CE exposed rat astrocytes. The activated effects of 2-CE on MAPK signal pathways were partially mediated by increased ROS, which was generated probably in the course of 2-CE metabolism.

## Author Contributions

TW carried out, analyzed experimental results, and wrote the manuscript. QS, HT, YL, FZ and GW edited the manuscript. YJ conceived the study, designed and interpreted experiments and revised the manuscript.

## Conflict of Interest Statement

The authors declare that the research was conducted in the absence of any commercial or financial relationships that could be construed as a potential conflict of interest.

## Abbreviations

1,2-DCE: 1,2-dichloroethane
2-CE: 2-chloroethanol
CYP2E1: cytochrome P450 2E1
ERK: extracellular signal regulated protein kinase
JNK: c-Jun N-terminal kinase
MAPK: mitogen-activated protein kinase
MMP-9: matrix metalloproteinase-9
NAC: n-acetyl-l-cysteine
ROS: reactive oxygen species.
